# Effectiveness of Forum Play to promote respectful maternity care: A pilot intervention investigating self-reported perception and behaviour among care providers in urban Nepal

**DOI:** 10.1371/journal.pone.0349437

**Published:** 2026-06-03

**Authors:** Narayani Paudel Ghimire, Pranab Dahal, Sunil Kumar Joshi, Katarina Swahnberg

**Affiliations:** 1 Department of Health Sciences, Linnaeus University, Kalmar, Sweden; 2 Department of Nursing, Kathmandu Medical College, Kathmandu, Nepal; 3 Department of Health and Caring Sciences, Linnaeus University, Kalmar, Sweden; 4 Department of Community Medicine, Kathmandu Medical College, Kathmandu, Nepal; University of Nigeria - Enugu Campus, NIGERIA

## Abstract

**Background:**

Ensuring respectful maternity care is crucial to promote positive childbirth experiences among women. Globally, 22–100% women report experiencing at least one form of disrespect and abuse during facility-based birth, with a study in Nepal reporting 100% prevalence. Thus, interventions like Forum Play are needed to promote respectful maternity care but remain scarce. Forum Play is a participatory theatre technique which encourages the participants to create scenes based on their own experiences illustrating a situation of oppression. This study aimed to assess the effectiveness of a pilot Forum Play intervention in promoting respectful maternity care by investigating self-reported perception and behaviour among care providers.

**Methods:**

A quasi-experimental pre–post study with a control group was conducted among doctors and nurses in two tertiary hospitals in Nepal. Participants involved in providing care or interacting with women during labour and delivery were purposively selected for the intervention and control groups. The intervention group comprised 25 care providers (14 nurses and 11 doctors), while the control group included 75 care providers (42 nurses and 33 doctors). Two half-day Forum Play workshops were conducted separately for nurses and doctors in the intervention hospital. Data were collected using a self-administered structured questionnaire at baseline and two months post-intervention in both groups; however, the design was not strictly parallel. In the control hospital, data were collected later at the same intervals to provide contextual comparison. Data were analysed using IBM statistical package for social sciences version 20. Descriptive statistics (frequency, percentage, mean and standard deviation) were used to measure the self-reported perception and behaviour on disrespect and abuse and perceptions of respectful maternity care. Mann Whitney U test was used to compare groups and Wilcoxon Signed Rank test was used to assess within-group changes before and after the intervention.

**Results:**

There were no differences in background characteristics between intervention and control groups (p=>0.05), nor in the overall disrespect and abuse of women at baseline (p = 0.09 to 0.35) whereas significant differences were observed at follow-up (p=<0.001 to 0.004, r = 0.33 to 0.61). There were no significant differences in perception scores towards respectful maternity care between the groups at baseline while significant differences were found at follow-up (z = −3.25, p = 0.001 and r = 0.32). The perception score was significantly higher in the intervention group after the intervention (z = −3.073, p = 0.002 and r = 0.61) while no significant difference was observed in the control group (z = −0.120, p = 0.90 and r = 0.05).

**Conclusion:**

Forum Play as a method of participatory intervention has the potential to enhance positive perception and behavioral change among care providers towards respectful maternity care. Scaling up the Forum Play intervention and systematically exploring women’s experiences are essential to determine its actual effectiveness.

## Introduction

Respectful maternity care (RMC) is a universal human right of all pregnant women [1]. RMC is “the care organized for and provided to all women in a manner that maintains their dignity, privacy, and confidentiality, ensures freedom from harm and mistreatment, and enables informed choice and continuous support during labour and childbirth” [2]. The accessibility of RMC is fundamental to promoting care-seeking behaviour and ultimately ensuring the health and well-being of mothers and their newborns. The disrespectful and abusive behaviour of care providers has been identified as one of the barriers to seeking care [3].

Seven key areas of disrespect and abuse in childbirth were defined by a landscape analysis carried out in 2010 as: “physical abuse, non-consented care, non-confidential care, non-dignified care, discrimination based on specific patient attributes, abandonment of care, and detention in facilities” [4]. From a systematic review conducted in 2015, a comprehensive classification of the mistreatment of women during childbirth was established which comprises “physical abuse, sexual abuse, verbal abuse, stigma and discrimination, failure to meet professional standards of care, poor rapport between women and providers, and health system conditions and constraints” [5]. Despite several interventions aiming to address the problem, disrespect and abuse of women during childbirth is prevalent worldwide [6]. As per the findings of studies conducted in numerous countries, 22%−100% women have reported at least one type of disrespect and abuse during facility-based births (Northern Ethiopia: 22%, Southwest Ethiopia: 91.7%, Pakistan: 97.7%, southeastern Nigeria: 98%, central Ethiopia and Eastern Nepal:100%) [7–12].

Scholars highlight the importance of moving beyond the mere measurement of the prevalence of disrespect and abuse to focus on mechanisms that strengthen accountability for RMC [13]. Evidence from a systematic review indicated that interventions designed to promote RMC are essential for improving the overall quality of maternal health services, with positive outcomes reported in Tanzania and Kenya following such initiatives [14]. Another review, which synthesized findings primarily from six sub-Saharan African countries, further demonstrated that multi-component interventions were particularly effective in reducing mistreatment and fostering RMC [15]. Women cared for by providers who had received training in compassionate and respectful care were more likely to report RMC [16,17]. Forum Play workshops have previously been implemented as an intervention to enhance care providers’ knowledge and foster behavioral change regarding abuse in health care in Sri Lanka and Sweden demonstrating promising outcomes [18,19].

To uphold women’s constitutional rights to safe motherhood and reproductive health, the Government of Nepal enacted the Safe Motherhood and Reproductive Health Rights Act in 2018, which explicitly recognizes RMC as a fundamental right and mandates its provision in both public and private health facilities [20]. However, only 17% of women in Nepal reported that they received RMC [21]. Based on the listed disrespect and abuse in the landscape analysis, there is a high prevalence of disrespect and abuse reported by women [12]. In Nepal, healthcare providers’ conditions to provide RMC are often hampered by high workload, lack of adequate resources and training, and structural as well as cultural barriers. A higher client-to-provider ratio was found to be associated with lower levels of RMC practice in southwestern Nepal [22]. Integrating Forum play into healthcare training in Nepal can serve as a valuable tool to enhance the delivery of RMC by addressing provider stressors, improving communication skills, and promoting empathetic interactions between healthcare providers and women. Health care providers play a major role in achieving RMC, but there is a lack of studies on the perspective of care providers as well as tested interventions to promote RMC in Nepal. The aim of this study was to assess the effectiveness of a pilot Forum Play intervention in promoting respectful maternity care by investigating self-reported perception and behaviour among care providers.

In November 2023, two half-day Forum Play workshops were conducted with the intervention group. Forum Play, a participatory theatre method developed in Sweden by Katrin Byréus [23] and inspired by the work of Brazilian theatre practitioner Augusto Boal [24], engages participants in creating scenes from their own experiences to illustrate situations of oppression. The intervention was grounded in the theoretical frameworks of Freire’s *Pedagogy of the Oppressed* [25] and Boal’s *Theatre of the Oppressed* [24], adapted to the healthcare context. In addition, *Sexual and Reproductive Health and Rights* (SRHR) [26] informed the overall conceptual framework of the study. Together, these perspectives emphasise critical reflection, participation, and empowerment in addressing oppressive practices within care settings. Guided by these principles, Forum Play provided a structured and participatory space for healthcare providers to examine power dynamics, recognise potential violations of SRHR, and rehearse more respectful, rights-based approaches to maternity care.

In each workshop, participants were invited to share their experiences of abuse of women during labour and delivery, enabling everyone to hear and reflect on one another’s stories and to engage with the sensitivity of the theme. From these shared accounts, two cases were selected for enactment through Forum Play. Participants were then randomly divided into two groups, each rehearsing for fifteen minutes before performing their skit. The plays depicted scenarios of disrespect and abuse during pregnancy and childbirth, with roles such as oppressor, oppressed, and bystander. The remaining participants, acting as ‘spect-actors’ (who both observe and actively engage in enacting scenarios) [24], observed closely and were encouraged to interrupt the performance whenever they perceived that abusive behaviour had occurred or was about to occur. They could then intervene by replacing the character, demonstrating abusive behaviour, enacting and testing alternative, respectful responses. These interventions included not only verbal strategies but also non-verbal gestures such as gentle touch or eye contact with the patient. Multiple alternatives were attempted, demonstrating how bystander engagement and different strategies could help counteract disrespect and abuse and promote respectful care.

The workshops were facilitated by an expert Swedish drama pedagogue, with additional support from two artists of Actors’ Studio Nepal (a local drama group) to exchange and strengthen local capacity in Forum Play.

## Methodology

### Study design and setting

A quasi-experimental pre–post study with a control group was conducted in the Nursing, Obstetrics/Gynaecology, Anaesthesiology, and Neonatology units in two tertiary hospitals in Kathmandu, Nepal, one as an intervention and another as a control site. Both hospitals are comparable in their organizational setup, with a similar number and composition of care providers. Approximately 200 professional doctors and 300 nurses are currently employed in both hospitals. On average, each hospital manages approximately 1,500 deliveries annually, including Cesarean sections. These two hospitals were chosen as they offer comparable settings while being sufficiently distant to avoid contamination between study samples.

### Study population and sampling

Nurses providing care during labour, delivery, and the postnatal period, and doctors (obstetricians/gynaecologists, anaesthesiologists, and neonatologists) involved directly or indirectly in maternity care with at least one year of work experience were purposively selected as study participants. Random sampling was not feasible due to the small number of eligible care providers within the selected hospitals. Therefore, all available and eligible healthcare providers meeting the inclusion criteria were included to ensure an adequate sample size for the pre–post analysis. Initial approval for the study was obtained from the hospital directors. Subsequently, potential participants were identified in consultation with the heads of departments for doctors and the hospital matrons for nurses.

### Sample size

We used G* Power 3.1 to estimate the sample size. Taking 0.63 effect size [27], with 80% power at 0.05 level of significance, considering the allocation of intervention: control group at 1:3 ratio, the calculated total sample size was 114 (29 in the intervention group and 85 in the control group). Adding a 5% non-response rate, the total calculated sample size was 120 (30:90). However, of the 30 participants (15 doctors and 15 nurses) who were invited to the intervention, only 25 (14 nurses and 11 doctors) took part in the intervention. So, the final sample size for the analysis of data was 100 (25:75) in the pre-test. Fortunately, there was no dropout in the post-test. Participants were divided into intervention and control groups as follows:

Intervention group: A total of 25 care providers, 14 Nurses and 11 Doctors (6 from Obstetrics/Gynaecology, 3 from Anesthesiology, and 2 from Neonatology)
Control group: A total of 75 care providers, 42 Nurses and 33 Doctors (18 from Obstetrics/Gynaecology, 9 from Anesthesiology, and 6 from Neonatology)

### Research tool

A structured questionnaire was developed based on an extensive literature review and authors’ experience. The questionnaire was divided into three parts. Details of the tool are presented in Table 1.

**Table 1:** Description of research tool.

Part	Content	Number of questions/ items	Source/Origin	Scoring/Analysis
I	Background characteristics of the participants	Seven questions	Author-developed	Frequency, percentage, mean, median
II	Care providers’ views and acting on disrespect and abuse* of women	Four questions	Studies conducted in Sri Lanka and Sweden [18,19]. The term Abuse in Health Care was replaced by disrespect and abuse of women to meet the objectives of the study.	Frequency, percentage, Fisher Exact test
III	Perception towards respectful maternity care	Five-point Likert Scale with 19 items (15 positive statement and four negative)	Students’ perception of respectful maternity care (SPRMC) developed, validated and used in Nepal [28], Development and psychometric properties of Midwives’ Knowledge and Practice Scale on Respectful Maternity Care, Iran (MKP-RMC) [29], and Midwives’ perspective of respectful maternity care during childbirth [30]. Also, author-developed based on experience	Each item’s score ranging from 1–5 1-Strongly disagree 2-Disagree 3-Neutral 4-Agree 5-Strongly agree Reverse coding for negative statement Minimum score 19 and maximum score 95 Mean ±SD calculation

*Disrespect and abuse include “physical abuse, non-consented care, non-confidential care, non-dignified care, discrimination based on specific patient attributes, abandonment of care, and detention in facilities during labour and childbirth”.

The tool was pre-tested among a 10% sample size of the intervention group, and a few necessary modifications were made. The Cronbach’s alpha for part II (views and acting on disrespect and abuse) question was 0.61 in pretest and 0.64 in posttest. Likewise, Cronbach’s alpha for perception towards respectful maternity care was 0.70 in pretest and 0.75 in posttest. A definition of disrespect and abuse was provided to participants as a note to enhance the objectivity of the part II questions (Table 1). The questionnaire was translated into Nepali and then independently back translated into English by subject experts to ensure accuracy and equivalence prior to pretesting. Data were collected using the Nepali version of the questionnaire, administered in a paper-and-pencil format.

### Data collection

A self-administered questionnaire was used in both groups to collect baseline and follow-up data. Follow-up data were collected after a two-month interval, which was selected to capture early changes in participants’ awareness, perceptions, and initial behavioural practices following the pilot Forum Play intervention. In the intervention group, baseline data were collected from 23 to 26 November 2023 and follow-up data were collected from 23 to 26 January 2024. In the control group, baseline data were collected from 20 to 25 January 2024 and follow-up data were collected from 20 to 25 March 2024. Data collection in the control hospital was conducted approximately two months after the intervention due to a delay in obtaining ethical approval at that site. The intervention had been scheduled six months in advance and could not be postponed because the drama pedagogue was available in Nepal for a limited period. While this resulted in a temporal difference between the two arms, the same study procedures, tools, and inclusion exclusion criteria were used in both hospitals to maintain comparability.

### Data analysis

Data were analysed using the IBM Statistical Package for Social Sciences (SPSS) version 20. Descriptive statistics used frequency and percentage to describe the care providers’ background characteristics and views on disrespect and abuse of women. Differences in demographic characteristics between the intervention and control groups were assessed using an independent t-test (for age), a Mann–Whitney U test (for work experience), and a chi-square test (for other variables). Considering the small sample size and purposive sampling, Fisher’s exact tests was used to identify differences in perception and behaviour on disrespect and abuse of women between the groups before and after the intervention.

Item wise mean perception towards RMC was calculated before and after the intervention. Since the data were not normally distributed, Mann Whitney U test was used to compare groups and Wilcoxon Signed Rank test was used to assess within-group changes in perception of RMC before and after the intervention. The Mann Whitney U test is a non-parametric statistical test used to compare differences between two independent groups when the dependent variable is ordinal or continuous but not normally distributed. The Wilcoxon signed-rank test is a non-parametric test used to compare two related samples, such as before–after measurement as an alternative to the paired t-test when the data are not normally distributed. A p-value of <0.05 was considered as statistically significant. Effect size, r was calculated to find the practical significance. A r value of ≥0.3 was considered a moderate effect size and ≥0.5 was considered a large effect size.

### Ethical considerations

We conducted this study in accordance with the ethical principles of the Declaration of Helsinki. Ethical approval for the study was obtained from the Institutional Review Committee of both hospitals (Ref. 29092023/01 and 42–080/081 intervention and control hospital respectively). Permission for data collection was granted by the authorities of the hospitals and written informed consent was taken from each participant before data collection. Each participant was provided a full explanation regarding the purpose and the procedure of the study. The involvement of the participants in the study was voluntary, and they were informed that they could interrupt their participation at any time without explaining. Participants were assured that the provided information would be kept confidential and used only for study purposes. To keep the information confidential, the name of hospitals has not been disclosed.

## Results

**Fig 1:**
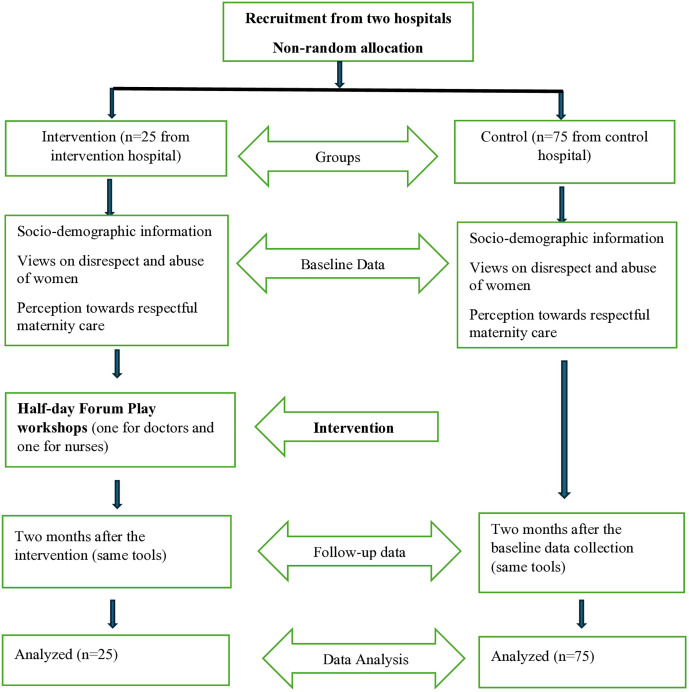
Flow diagram outlining the study process. Participants were divided into intervention and control groups. Forum Play workshops were conducted as intervention.

A total of 100 participants were recruited for the study in baseline, and all were followed-up after a two-month interval. There was no sample attrition in the follow-up data collection. The process of recruitment and data collection in the study has been displayed in Fig 1.

### Background characteristics of the participants

The mean age of care providers was 31.1 years in the intervention group and 31.4 years in the control group. No statistically significant differences were found between the groups with regards to socio-demographic variables [age (p = 0.87), sex (p = 0.37), ethnicity (p = 0.41), marital status (p = 0.80), education (p = 0.45), occupation (1.00) and work experience (p = 0.09)]. The background characteristics of the groups are illustrated in Table 2.

**Table 2:** Background characteristics of the participants (N = 100).

Variables	Intervention (n = 25) n (%)	Control (n = 75) n (%)	*p*-value
**Age (in years)**			
Mean ± SD	31·1 ± 4·11	31·4 ± 8·94	0·87^
**Sex**			
Male	5 (20.0)	11 (14·7)	0·37
Female	20 (80.0)	64 (85·3)	
**Ethnicity**			
Brahmin & Chhetri	10 (40.0)	37 (50)	0·41
Janajati	12 (48.0)	32 (42)	
Madheshi	3 (12.0)	6 (8.0)	
**Marital status**			
Married	14 (56.0)	41 (54.0)	0·80
Unmarried	11 (44.0)	34 (46.0)	
**Highest education**			
Diploma	4 (16.0)	20 (26·7)	0·45
Bachelor	10 (40.0)	22 (29·3)	
Masters	11 (44.0)	33 (44.0)	
**Occupation**			
Doctor	11 (44.0)	33 (44.0)	1·00
Nurse	14 (56.0)	42 (56.0)	
**Work Experience (in years)**			
Median (IQR)	5 (2-10)	2 (1-9)	0·09#

^t-test, # Mann Whitney U test, IQR = Interquartile range.

### Perceptions and behaviour on disrespect and abuse of women

Responses regarding perception and behaviour on disrespect and abuse of women during facility-based birth changed significantly in the intervention group after the intervention, while no such changes were observed in the control group. In the intervention group, the proportion of participants who reported hearing about disrespect and abuse in their workplace increased from eight (32%) before to 14 (56%) after the intervention. At baseline, most participants reported not having been exposed to such situations [21 (84%) in the intervention group and 55 (73.3%) in the control group]. After the intervention, however, only nine (36%) of the intervention group reported no exposure.

Regret for not acting against disrespect and abuse was also more common after the intervention: 10 (40%) of the intervention group expressed regret compared to none before the intervention, whereas only 4% of the control group expressed such regret at baseline. Importantly, no participant in either group reported personally engaging in disrespect or abuse even after the intervention.

At baseline, there was no statistically significant difference in perception and behaviour on disrespect and abuse of women between the groups (p = 0.09–0.35). By follow-up, however, significant differences were observed (p < 0.001–0.004) with moderate to large effect sizes (0.33–0.61) (Tables 3 and 4).

**Table 3:** Baseline perception and behaviour on disrespect and abuse of women (N = 100).

Questions	Options	Intervention (25) n (%)	Control (75) n (%)	*p*-value #
How is disrespect and abuse of women of relevance to you?	No relevance	5 (20.0)	33 (44.0)	0·09
	Picked up some general aspects about disrespect and abuse of women	12 (48.0)	24 (32.0)	
	Heard about cases of disrespect and abuse of women at my workplace	8 (32.0)	18 (24.0)	
	Have been involved personally in cases of disrespect and abuse of women	–	–	
Can you recall your response in a situation of disrespect and abuse women?	Have not been in such a situation	20 (80.0)	53 (70·5)	0.35
	I had no possibility to respond in any way	–	9 (12.0)	
	I acted supporting my colleagues’/staff’s position	1 (4.0)	2 (2·7)	
	I acted supporting the patient’s position	4 (16.0)	11 (14·7)	
Have you experienced regret in this situation?	Have not been in such a situation	21 (84.0)	55 (73·3)	0.14
	No	3 (12.0)	17 (22·7)	
	Yes, about not acting against disrespect and abuse of women	–	3 (4.0)	
	Yes, about acting on behalf of the staff.	–	–	
	Yes, about acting on behalf of the patient	1 (4.0)	–	
How important is it to consider the patient’s perspective in Obs/Gyn* in relation to the medical perspective?	Not so important	–	–	0.25
	Little important	1 (4.0)	–	
	Very important	24 (96.0)	75 (100)	

*Obstetrics/Gynaecology, # Fisher Exact test.

**Table 4:** Follow-up perceptions and behaviour on disrespect and abuse of women (N = 100).

Questions	Options	Intervention (25) n (%)	Control (75) n (%)	*p*-value #/ Effect size
How is disrespect and abuse of women of relevance to you?	No relevance	4 (16.0)	35 (46·7)	0·004*/0.33
	Picked up some general aspects about disrespect and abuse of women.	7 (28.0)	23 (30·6)	
	Heard about cases of disrespect and abuse of women at my workplace	14 (56.0)	17 (22·7)	
	Have been involved personally in cases of disrespect and abuse of women	–	–	
Can you recall your response in a situation of disrespect and abuse of women?	Have not been in such a situation	12 (48.0)	67 (89·4)	<0·001*/ 0.44
	I had no possibility to respond in any way	6 (24.0)	4 (5·3)	
	I acted supporting my colleagues’/staff’s position	1 (4.0)	–	
	I acted supporting the patient’s position	6 (24.0)	4 (5·3)	
Have you experienced regret in this situation?	Have not been in such a situation	9 (36.0)	68 (90·7)	<0·001*/ 0.61
	No	5 (20.0)	6 (8.0)	
	Yes, about not acting against disrespect and abuse of women	10 (40.0)	1 (1·3)	
	Yes, about acting on behalf of the staff.	1 (4.0)	–	
	Yes, about acting on behalf of the patient	–	–	
How important is it to consider the patient’s perspective in Obs/Gyn in relation to the medical perspective?	Not so important	–	–	··
	Little important	–	–	
	Very important	25 (100)	75 (100)	

··not applicable, # Fisher Exact test * Statistically significant at p value <0.05.

### Perception towards respectful maternity care

Although participants in both groups generally reported positive perceptions of RMC, negative perceptions persisted in key areas (mean score <4), particularly regarding labor companionship and the women’s preferred birth positions (Table 5). At follow-up, median perception scores increased in the intervention group but decreased in the control group (Table 6).

**Table 5:** Item wise mean perception score towards respectful maternity care.

S. N.	Respectful Maternity Care Aspects (Items)	Intervention group		Control group	
		Pretest Mean ± SD	Posttest Mean ± SD	Baseline Mean ± SD	Follow-up Mean ± SD
1	Care providers should welcome laboring woman warmly and introduce themselves	4·68 ± 0·47	4·60 ± 0·57	4·37 ± 0·81	4·32 ± 0·73
2	Establishing a good and friendly relationship is essential for providing respectful maternity care.	4·68 ± 0·55	4·60 ± 0·91	4·47 ± 0·75	4·20 ± 0·97
3	The laboring woman should not allow to have companion inside the labor and delivery room*	3·84 ± 1·10	3·60 ± 1·08	3·73 ± 0·81	3·51 ± 0·96
4	Unnecessary interventions without medical indication should be eliminated (e.g., oxytocin, episiotomy, CS)	3·64 ± 1·28	4·12 ± 0·66	4·03 ± 0·97	4·03 ± 0·98
5	Women need to get information about progress of labor	4·52 ± 0·51	4·76 ± 0·43	4·58 ± 0·55	4·45 ± 0·82
6	All interventions should be performed with laboring woman’s informed consent.	4·60 ± 0·57	4·60 ± 0·50	4·64 ± 0·47	4·36 ± 0·89
7	There should be provision of drug-free comfort and pain relief methods during labor	4·08 ± 0·99	4·32 ± 0·69	3·80 ± 0·71	3·75 ± 1·06
8	Care providers should pay attention to laboring women’s safety in providing care and doing interventions.	4·52 ± 0·58	4·72 ± 0·45	4·64 ± 0·62	4·45 ± 0·81
9	Women should be encouraged to actively participate in their care.	4·64 ± 0·49	4·44 ± 0·50	4·56 ± 0·68	4·40 ± 0·80
10	Preserving dignity is preventing any mistreatments with laboring women.	4·32 ± 0·90	4·24 ± 0·66	4·12 ± 1·02	4·05 ± 0·94
11	Care providers should keep all medical records of women confidential.	4·00 ± 0·95	4·48 ± 0·77	4·24 ± 0·92	4·15 ± 1·00
12	Equal care should be provided to all women, regardless of their ethnicity, culture, religion, etc.	4·76 ± 0·66	4·64 ± 0·56	4·69 ± 0·63	4·53 ± 0·81
13	Care providers should be continuously or timely available during labor and delivery.	4·80 ± 0·40	4·44 ± 0·65	4·53 ± 0·64	4·39 ± 0·80
14	It is often hard to keep a clean and calm environment for women during labour *	2·64 ± 1·25	2·52 ± 1·12	2·45 ± 1·01	2·56 ± 1·16
15	Care providers should try to limit exposing a woman’s body when providing care.	4·28 ± 1·06	4·40 ± 0·57	4·20 ± 0·54	4·32 ± 0·75
16	Care providers should support laboring woman to be in her desired birthing position.	3·28 ± 1·13	3·64 ± 0·95	3·53 ± 0·97	3·68 ± 1·10
17	Care providers may beat the laboring woman if she does not cooperate*	4·44 ± 0·87	4·56 ± 1·12	4·41 ± 0 ∙ 88	4 ∙ 31 ± 1 ∙ 05
18	Care providers may shout at laboring woman if she does not cooperate*	3 ∙ 92 ± 1 ∙ 22	4 ∙ 52 ± 0 ∙ 91	3 ∙ 95 ± 1 ∙ 11	4 ∙ 05 ± 1 ∙ 17
19	Care providers should provide evidence-based and up-to-date childbirth care	4 ∙ 40 ± 0 ∙ 76	4 ∙ 56 ± 0 ∙ 50	4 ∙ 41 ± 0 ∙ 57	4 ∙ 29 ± 0 ∙ 80

*Reversed score, the maximum possible score of each item is 5, mean ≥ 4 implies positive perception.

**Table 6:** Difference between the groups regarding perception towards respectful maternity care before and after the intervention.

Data timing	Median (IQR) perception		U	Z	*p*-value	Effect size
	Intervention (n = 25)	Control (n = 75)				
Baseline	81 (74-86)	80 (76-84)	922.50	−0.120	0.90	0·01
Follow up	86 (76-86)	78 (73-85)	529.50	−3·253	**0.001**	**0.32**

A Mann–Whitney U test showed no significant difference between groups at baseline (z = −0.120, p = 0.90, r = 0.01), but a significant difference at follow-up (z = −3.25, p = 0.001, r = 0.32). Similarly, the Wilcoxon signed-rank test (Table 7) revealed a significant improvement in the intervention group from baseline to follow-up (z = −3.073, p = 0.002, r = 0.61), while no significant change was observed in the control group (z = −0.120, p = 0.90, r = 0.05).

**Table 7:** Comparison of perception score towards respectful maternity care before and after the intervention using Wilcoxon signed-rank test.

Group	Median (IQR) perception		Z	*p*-value	Effect size
	Pretest	Posttest			
Intervention (n = 25)	81 (74-86)	86 (76-86)	−3.073	**0.002**	**0.61**
Control (n = 75)	80 (76-84)	78 (73-85)	−0.454	0.65	0.05

## Discussion

Disrespect and abuse during facility-based birth is a form of abuse in health care [31–33]. This study evaluated the effectiveness of Forum Play in training care providers to recognize and respond to such behaviors and to promote respectful maternity care. Findings showed significant changes in the intervention group, with more participants reporting awareness of disrespect and abuse in their workplace after the intervention (56% vs. 32% before). Additionally, 40% expressed regret for not acting against such situations after the intervention, compared to none before. These shifts were not observed in the control group. Similar results have been reported in Sweden, where Forum Play workshops improved staff members’ ability to act in situations of abuse in health care [19]. Strengthening providers’ capacity to recognize and address disrespect and abuse is critical, as this directly contributes to women’s likelihood of receiving RMC [34].

A study in Sri Lanka using Forum Play with maternity staff found that participants later reported more frequent involvement in situations of disrespect and abuse of women [18]. In contrast, no participants in our study admitted personal involvement after the intervention, even though some had shared such experiences during the workshops. This difference may reflect contextual factors in Nepal, where admitting involvement in disrespectful care carries stigma or fear of professional repercussions, most likely leading to underreporting. While Forum Play provides a safe space for reflection, translating this into honest acknowledgment remains challenging. Interventions should therefore address both awareness and the cultural or structural barriers that hinder open reporting of mistreatment.

The findings of this study can be interpreted in relation to the theoretical frameworks underpinning the intervention. SRHR provided the overarching lens, emphasising dignity, autonomy, and the right to respectful maternity care [26]. Freire’s *Pedagogy of the Oppressed* contributed the concept of critical consciousness; whereby healthcare providers are encouraged to reflect on and question normalized practices that perpetuate disrespect and abuse [25]. Similarly, Boal’s *Theatre of the Oppressed* informed the use of experiential and participatory learning through role-play, enabling participants to rehearse alternative actions and challenge power hierarchies in a safe environment [24]. Together, these frameworks helped explain the observed improvements in awareness, perceptions, and willingness to act, as they fostered reflection, dialogue, and embodied learning beyond traditional didactic training approaches.

Overall, participants in both groups reported positive perceptions of RMC, consistent with findings from Nigeria where providers generally held favorable views [35]. In our study, providers strongly endorsed principles such as obtaining informed consent, ensuring women’s safety during care, and providing equal treatment regardless of background. Importantly, the intervention group showed an increase in mean perception scores at follow-up, echoing results from Kenya where similar training enhanced providers’ recognition of women’s rights, including informed consent privacy, and dignity [36]. These findings suggest that while baseline perceptions of respectful care may be positive, participatory interventions can strengthen providers’ awareness and commitment towards RMC.

In our study, negative perceptions persisted in certain areas, such as allowing birth companions and supporting the women’s preferred birth positions, despite the intervention. In contrast, midwives in Zimbabwe demonstrated improved women-centered practices following RMC training [37]. This suggests that combining Forum Play with targeted training on specific components of RMC may be necessary to strengthen providers’ positive perceptions and support for these practices in the Nepalese context. Moreover, structural constraints such as workload, resource limitations, and working conditions significantly influence providers’ ability to implement RMC. Therefore, interventions should not focus solely on individual behaviour change but should also address systemic barriers to achieve sustainable improvements in RMC.

At baseline, there were no statistically significant differences regarding views on disrespect and abuse of women between intervention and control groups whereas significant differences were observed at follow-up. This indicates that Forum Play has the potential to engage and train the care providers to not only increase awareness but also act against disrespect and abuse of women during facility-based birth. Interventions that encourage active participation, role-play, and reflection as Forum Play are found to be particularly effective in shifting mindsets compared to didactic approaches. Furthermore, raising awareness at the provider level creates a foundation for broader systemic change, as sensitized providers are more likely to advocate for respectful practices within their institutions. The findings are consistent with the study done in Sri Lanka, which reported that the intervention enhanced the abilities of health care providers to recognize disrespect and abuse of women, an essential first step in reducing such practices [18]. In contrast, the study among maternity care providers in Sweden could not confirm an increased awareness of abuse in health care [19], possibly due to differences in setting, cultural context, or baseline levels of awareness.

In our study, no significant difference was observed in the median perception score towards RMC between intervention and control groups at baseline; however, a significant difference emerged at follow-up. The intervention group showed a higher median perception score with a large effect size (z = −3.073, p = 0.002, r = 0.61). This finding aligns with a quasi-experimental study among nursing students in Nepal, where online education significantly improved positive perceptions of RMC [38]. Similarly, a mixed-methods systematic review reported that educational interventions enhanced knowledge and perceptions of respectful care while reducing women’s experience of mistreatment [39]. These findings suggest that targeted interventions are effective in shaping providers’ attitudes and awareness regarding RMC. Improved perceptions among healthcare providers are important because they represent a critical first step towards fostering behavioral change in clinical practice.

### Strengths and limitations

This pilot intervention study on RMC using Forum Play represents a novel and innovative approach within the hierarchical clinical context of Nepal. Alongside doctors and nurses, baseline and follow-up data were initially sought from administrative staff through a third Forum Play workshop. Although all participants showed strong motivation to engage, the control hospital authority considered the topic highly sensitive and initially resisted granting permission for data collection. After consultation with the relevant authority, approval was obtained to conduct data collection approximately two months later than in the intervention hospital and to include only doctors and nurses, excluding administrative staff. The final analysis was therefore limited to care providers. Although identical procedures, tools, and participant groups were used to enhance comparability, the non-parallel timing of data collection may have introduced unmeasured contextual influences on the findings. The relatively short two-month follow-up period limits the ability to assess long-term behavioural change. Nevertheless, as this study formed part of a pilot Forum Play intervention, a subsequent follow-up assessment was conducted one year after the intervention to explore the sustainability of changes over time.

These findings must be interpreted in light of several methodological constraints. The study involved a small sample size across only two hospitals (one intervention and one control), with the control site hesitant to participate fully. There was no random sampling, and the study did not control potential confounders or account for clustering effects. For example, there is difference in median work experience between the groups which may affect the exposure to disrespect and abuse and RMC. The study measured providers’ self-reported perceptions, which may not directly reflect their actual behaviors in clinical practice, particularly in high-stress situations such as staffing shortages, stockouts, high delivery volume, interpersonal issues, communication challenges, and the stress of dealing with obstetric emergencies. The absence of women’s perspective limits our ability to assess the intervention’s impact on patient outcomes and experiences. The moderate Cronbach’s alpha observed for part II of research tool indicates that further refinement and validation is needed. In addition, reliance on self-administered questionnaires may have introduced information bias, as responses could have been influenced by social desirability and participants’ varying levels of understanding.

### Direction of future work

Based on this pilot intervention, there are a qualitative study conducted among doctors, nurses, and administrative staff [Promoting respectful maternity care in Nepal: A qualitative exploratory study of a pilot Forum Play intervention among hospital staff] and a mixed method study [Evaluation of a pilot study promoting respectful maternity care: A one-year follow-up reflection on a Forum Play intervention among hospital staff in Nepal]. This is a small-scale study using purposive sampling. Although our results are promising and can form the platform for future randomized control trials, this intervention needs to be conducted on a large scale using random sampling with a long-term follow-up to further explore the effectiveness. Future research should include women’s perspectives to provide a more comprehensive evaluation of the intervention and its effects on respectful maternity care.

## Conclusion

Self-reported perceptions and behaviors of care providers improved in the intervention group after the training. Forum Play, by rehearsing real-life situations, proved to be a promising approach for raising awareness and fostering behavior change. Our findings indicate that it can effectively promote respectful maternity care in Nepal by preparing care providers to prevent disrespect and abuse during labor and delivery. However, the sample size was very small, and the study did not rely on direct observation of actual behavior change. To more rigorously establish the effectiveness of the Forum Play intervention, it should be implemented on a larger scale across maternity care settings, with exploration of women’s experience.

## Supporting information

S1 File Checklist. (DOCX)

S2 File Research questionnaire. (DOCX)

S3 File Pretest data file. (SAV)

S4 File Posttest data file. (SAV)
