# Supplementary material for: Effectiveness of Forum Play to promote respectful maternity care: A pilot intervention investigating self-reported perception and behaviour among care providers in urban Nepal
Source: PLoS One. 2026 Jun 3;21(6):e0349437. doi: 10.1371/journal.pone.0349437 (PMC13232825; doi:10.1371/journal.pone.0349437)
Supplement: S1 File — (DOCX) [file pone.0349437.s001.docx]

**Inclusivity in global research**

PLOS’ policy on inclusivity in global research aims to improve transparency in the reporting of research performed outside of researchers’ own country or community and ensures that PLOS publications reporting global research adhere to high standards for research ethics and authorship. Authors of relevant research articles may be asked to complete the questionnaire below, which outlines ethical, cultural, and scientific considerations specific to inclusivity in global research. This questionnaire may be requested when researchers have travelled to a different country to conduct research, if research uses samples collected in another country, research with Indigenous populations or their lands, or if research is on cultural artefacts. Researchers travelling to another country solely to use laboratory equipment will not normally be required to complete the questionnaire. However, the questionnaire can be requested at the journal’s discretion for any submission – if you have been requested to complete this questionnaire by the PLOS journal you submitted to, please do so.

Please complete the questionnaire below and include this as a Supporting Information file with your manuscript. Note that if your paper is accepted for publication, this checklist will be published with your article in the supporting information files. Please ensure that you reference the checklist in the main body of your manuscript. We suggest adding a subsection ‘Inclusivity in global research’ to your Methods section and adding the following sentence: “Additional information regarding the ethical, cultural, and scientific considerations specific to inclusivity in global research is included in the Supporting Information (SX Checklist)”

The questions have been designed to be applicable to a wide range of study types, and there are subsections for both human subjects research and non-human subjects research. If any of the questions are not relevant to your research, please mark them as “N/A” as appropriate.

**Ethical considerations, permits and authorship**

*This section is applicable to all research types.*

Provide details as to who granted permission and/or consent for the study to take place in the Methods section of your manuscript. This should include the names of **all** ethics boards, governmental organizations, community leaders or other bodies that provided approval for the study. If individuals provided approval refer to these people by their role or title but do not list their name(s).

Yes, ethical approval for the study was obtained from the Institutional Review Committee of both hospitals (Ref. 29092023/01 and 42-080/081 intervention and control hospital respectively). To keep the information confidential, the name of hospitals has not been disclosed.

This is reported in methodology under ethical considerations.

If there were any deviations from the study protocol after approval was obtained, please provide details of these changes in the Methods section of your manuscript.

N/A

Did this study involve local collaborators that are residents of the country where the research was conducted, or members of the community studied? If you do not have any authors from said communities, please provide an explanation for this below.

Yes, this study involved local collaborators who are residents of Nepal, where the research was conducted. Three out of the four authors of this manuscript are residents of Nepal. These authors include the first author, who is PhD candidates, second and third authors serve as local supervisors providing crucial insights and guidance throughout the research process.

Everyone listed as an author should meet PLOS’ criteria for authorship and all individuals who meet these criteria should be included in the author byline, rather than the acknowledgements. For further information please see the journal’s Authorship Policy.

**Human subjects research (e.g. health research, medical research, cross-cultural psychology)**

Did you obtain written informed consent from a representative of the local community or region before the research took place? How did you establish who speaks for the community? Details of written informed consent obtained from study participants should be reported separately in the Methods section of your manuscript.

Permission for data collection was granted by the authorities of the hospitals and written informed consent was taken from each participant before data collection. Each participant was provided a full explanation regarding the purpose and the procedure of the study. The involvement of the participants in the study was voluntary and they were informed that they could interrupt their participation at any time without explaining. Participants were assured that the provided information would be kept confidential and used only for study purposes.

This is reported in methodology under ethical considerations.

How did members of the local community provide input on the aims of the research investigation, its

methodology, and its anticipated outcome(s)?

We organized a meeting with hospital principals, Directors, Heads of the Departments, Hospital Matrons to inform them about the research project, at both intervention and control site. The authorities of Intervention site facilitated us to conduct interventional workshops and to collect data. The authorities of control site helped us to collect data by granting the permission.

When engaging with the local community, how did you ensure that the informed consent documents and other materials could be understood by local stakeholders?

The informed consent document was written in straightforward, local language that was easily understood by community members. We conducted pretesting of the consent materials to find out its clarity. Additionally, we provided verbal explanations of the consent documents along with written materials to ensure participant comprehension. Participants have noted that the written materials are clear, relevant. They do not raise any questions about their intended meaning.

Will the findings of the research be made available in an understandable format to stakeholders in the community where the study was conducted (e.g. via a presentation, summary report, copies of publications, etc.)? Please provide details of how this will be achieved.

Yes, we will prepare reports of the key findings for local stakeholders (the two participating hospitals). The final PhD thesis dissertations resulting from this project will be shared at a dissemination conference, where we will invite and share key findings with hospital staff, and department personnel. We will also share the link of article after publication.

**Non-human subjects research using specimens/ animals collected as part of the study, or those housed in archival collections. Examples include archaeology, paleontology, botany and zoology.**

Did the permission you obtained from a local authority to perform the study include an agreement on access to outputs and benefit sharing? This may include procedures to enable fair distribution of the benefits and resources arising from the research performed. Please include any details of Prior Informed Consent and Benefit Sharing Agreements obtained. These may be required by field-specific regulations, for example the Convention on Biological Diversity (CBD) and the associated Nagoya Protocol.

N/A

If the material used in your study was imported, please A) provide the year it was imported and B) indicate whether permits were obtained to import/export the materials used, C) provide details of any permits obtained. If this information is not available, please indicate this.

N/A

If you used archival specimens, please state how the material used in your study was acquired by the institute it is held in and provide details of any permits obtained for the original excavations/ sample collection. If this information is not available, please indicate this.

N/A

How was the potential cultural significance of the materials collected in your study to local communities considered in your research design? Were Indigenous peoples and/or local researchers and institutions involved with archaeological excavations / collection of specimens? If so, please provide a description of their involvement.

N/A

If your manuscript includes photographs of human remains please indicate whether authors obtained permission from descendants or affiliated cultural communities to do so.

N/A
