# Supplementary material for: Effectiveness of Forum Play to promote respectful maternity care: A pilot intervention investigating self-reported perception and behaviour among care providers in urban Nepal
Source: PLoS One. 2026 Jun 3;21(6):e0349437. doi: 10.1371/journal.pone.0349437 (PMC13232825; doi:10.1371/journal.pone.0349437)
Supplement: S2 File — (DOCX) [file pone.0349437.s002.docx]

**Research questionnaire**

**Part I: Socio-demographic information (Please √ into the appropriate box)**

1. Age (Completed years) ……
2. Sex:

- Male
- Female

1. Ethnicity:

- Brahmin/Chhetri
- Janjati
- Dalit
- Madhesi
- Others (Please specify) ……….

1. Marital status:

- Unmarried
- Married
- Others (Please specify) ………………….

1. Profession

- Doctor
- Nurse
- Administrative staff

1. Highest educational qualification (Please √ your highest degree)

- Basic education (up to 8 Class)
- Secondary education (9 -12 Class)
- Certificate level
- Bachelor
- Postgraduate student
- Master
- DM

1. Department:

- Nursing
- Obstetrics/Gynaecology
- Anaesthesiology
- Neonatology
- Administration

1. Working experience (Please write your work experience in years): ……………

**Part II: Staff’s views and acting on disrespect and abuse of women during institutional delivery**

**Instructions:** Please place a check mark (√) in one of the options for each question. Please choose the option based on your current (in this hospital) as well as previous experience (previous hospital where you worked)

| ***Note:*** *Disrespect and abuse include “physical abuse, non-consented care, non-confidential care, non-dignified care, discrimination based on specific patient attributes, abandonment of care, and detention in facilities during labour and childbirth”.*   1. How is disrespect and abuse of women relevance to you?  - No Relevance - Picked up some general aspects about disrespect and abuse of women. - Heard about cases of disrespect and abuse of women.at my workplace. - Have been Involved personally in cases of disrespect and abuse of women.  1. Can you recall your response in situation of disrespect and abuse of women?  - Have not been in such a situation. - I had no possibility of responding in any way. - I acted supporting my colleagues'/staff's position. - I acted supporting the patient's position.   .   1. If you have in such a situation, have you experienced regrets in this situation?  - No - Yes, about not acting against disrespect and abuse of women. - Yes, about acting on behalf of the staff. - Yes, about Acting on behalf of the patient.  1. Did you act in agreement with? **(More than one option can be chosen)**  - Universal Declaration of Human Rights - World Medical Association (WMA) ethical guidelines - International Federation of Gynecology and Obstetrics (FIGO) ethical guidelines - International Council of Nurses (ICN) code of ethics for nurses - International Confederation of Midwives (ICM) International Code of Ethics for   Midwives   - I don’t know.  1. How important is it to consider the patient's perspective in obstetrics/gynecology in relation to the medical perspective?  - Not so important - Little important - Very important |
| --- |

**Part III: Perception of care providers towards respectful maternity care**

**Instruction:** Please indicate how strongly you agree or disagree with each of the following statements by placing a check mark (√) in one of the columns of each item. Please complete every item.

| **S.**  **N.** | **Respectful Maternity Care Aspects (Items)** | **SD (1)** | **D (2)** | **N (3)** | **A (4)** | **SA (5)** |
| --- | --- | --- | --- | --- | --- | --- |
| 1 | Care providers should welcome laboring woman warmly and introduce themselves |  |  |  |  |  |
| 2 | Establishing a good and friendly relationship is essential for providing respectful maternity care. |  |  |  |  |  |
| 3 | The laboring woman should not allow to have companion inside the labor and delivery room. |  |  |  |  |  |
| 4 | Unnecessary interventions without medical indication should be eliminated (e.g., oxytocin, episiotomy, CS) |  |  |  |  |  |
| 5 | Women need to get information about progress of labor |  |  |  |  |  |
| 6 | All interventions should be performed with laboring woman’s informed consent. |  |  |  |  |  |
| 7 | There should be provision of drug-free comfort and pain relief methods during labor |  |  |  |  |  |
| 8 | Care providers should pay attention to laboring women’s safety in providing care and interventions. |  |  |  |  |  |
| 9 | Women should be encouraged to actively participate in their care. |  |  |  |  |  |
| 10 | Preserving dignity is preventing any mistreatments with laboring women. |  |  |  |  |  |
| 11 | Care providers should keep all medical records of women confidential. |  |  |  |  |  |
| 12 | Equal care should be provided to all women, regardless of their ethnicity, culture, religion, etc. |  |  |  |  |  |
| 13 | Care providers should be continuously or timely available during labor and delivery. |  |  |  |  |  |
| 14 | It is often hard to keep a clean and calm environment for women during labour |  |  |  |  |  |
| 15 | Care providers should try to limit exposing a woman’s body when providing care. |  |  |  |  |  |
| 16 | Care providers should support laboring woman to be in her desired birthing position. |  |  |  |  |  |
| 17 | Care providers may beat the laboring woman if she does not cooperate. |  |  |  |  |  |
| 18 | Care providers may shout at laboring woman if she does not cooperate. |  |  |  |  |  |
| 19 | Care providers should provide evidence-based and up-to-date childbirth care |  |  |  |  |  |

**Note:** SD= Strongly disagree, D=Disagree, N=Neutral, A=Agree, SA=Strongly agree
